# Analysis of related factors of plasma antibody levels in patients with severe and critical COVID-19

**DOI:** 10.1038/s41598-024-52572-9

**Published:** 2024-01-31

**Authors:** Yudi Xie, Yang Xia, Haixia Xu, Jue Wang, Wei Zhang, Ling Li, Zhong Liu

**Affiliations:** 1 Institute of Blood Transfusion, Chinese Academy of Medical Sciences and Peking Union Medical College, 26 Huacai Road, Longtan Industry Zone, Chenghua District, Chengdu, Sichuan Province People’s Republic of China; 2 Key Laboratory of Transfusion Adverse Reactions, CAMS, Chengdu, Sichuan Province People’s Republic of China; 3 Department of Respiratory and Critical Care Medicine, Shanghai Changhai Hospital, The First Affiliated Hospital of Second Military Medical University, Shanghai, People’s Republic of China; 4 Department of Blood Transfusion, The Third People’s Hospital of Chengdu, Affiliated Hospital of Southwest Jiaotong University, 82 Qinglong Street, Qingyang District, Chengdu, Sichuan Province People’s Republic of China; 5 College of Public, Hygiene of Anhui Medical University, Hefei, Anhui Province People’s Republic of China

**Keywords:** Medical research, Immunology, Adaptive immunity, Infection, Infectious diseases, Vaccines

## Abstract

Coronavirus disease 2019 (COVID-19) continues to impact global public health. The severe acute respiratory syndrome coronavirus 2 (SARS-CoV-2) has become less virulent as it mutates, prompting China to ease restrictions at the end of 2022. With the complete reopening, a surge in COVID-19 cases has ensued. Therefore, we conducted a study to explore the correlation between plasma antibody levels and baseline conditions or clinical outcomes in severe and critical patients. We collected the basic information of 79 included patients. Enzyme-linked immunosorbent assay (ELISA) tests were performed on plasma samples. The receptor-binding domain (RBD) IgG antibody level of the mild group was significantly higher than that of the severe/critical group (*P* = 0.00049). And in the severe/critical group, there existed an association between plasma antibody levels and age (*P* < 0.001, r = − 0.471), as well as plasma antibody levels and vaccination status (*P* = 0.00147, eta^2^ = 0.211). Besides, the level of plasma antibody seemed to be moderately correlated with the age, indicating the need for heightened attention to infections in the elderly. And plasma antibody levels were strongly associated with vaccination status in the severe/critical patients.

## Introduction

Coronavirus disease 2019 (COVID-19), which was caused by severe acute respiratory syndrome coronavirus 2 (SARS-CoV-2), continues to influence global public health^1^. With the adaptive mutation of the virus, the virulence of the virus has gradually weakened^2^, and the country had constantly adjusted the corresponding prevention and control measures^3^. The epidemic in China was declared under control on December 7, 2022. Following the complete reopening of the country, a surge in SARS-CoV-2 infections has occurred. It is estimated that between December 2022 and February 2023, more than 82% of the country's population became infected with SARS-CoV-2, which means that about 1.15 billion people were infected with COVID-19^4^. The prevalent strain during that period was Omicron BA.4/5. Infected individuals can be divided into mild, severe, and critical patients according to their clinical symptoms. Additionally, severe and critical COVID-19 patients necessitated hospitalization, whereas mild patients did not. A significant correlation existed between disease progression and patients’ antibody levels^5^. And several studies have linked antibody levels to factors such as age and gender^6,7^. In addition, the vaccination of COVID-19 vaccine in China has been ongoing. To enhance the protective effect of the vaccine and increase the rapid growth of antibodies in the body, the government encouraged healthy individuals to get another dose of COVID-19 vaccine, called a booster shot, 6 months after the completion of routine vaccination. In this study, we evaluated the receptor-binding domain (RBD) IgG antibody titers of plasma samples collected from COVID-19 patients after the cancellation in China. We aimed to explore whether plasma antibody levels are correlated with baseline conditions and patients’ clinical outcomes in hospitalized patients.

## Results

**Figure 1:**
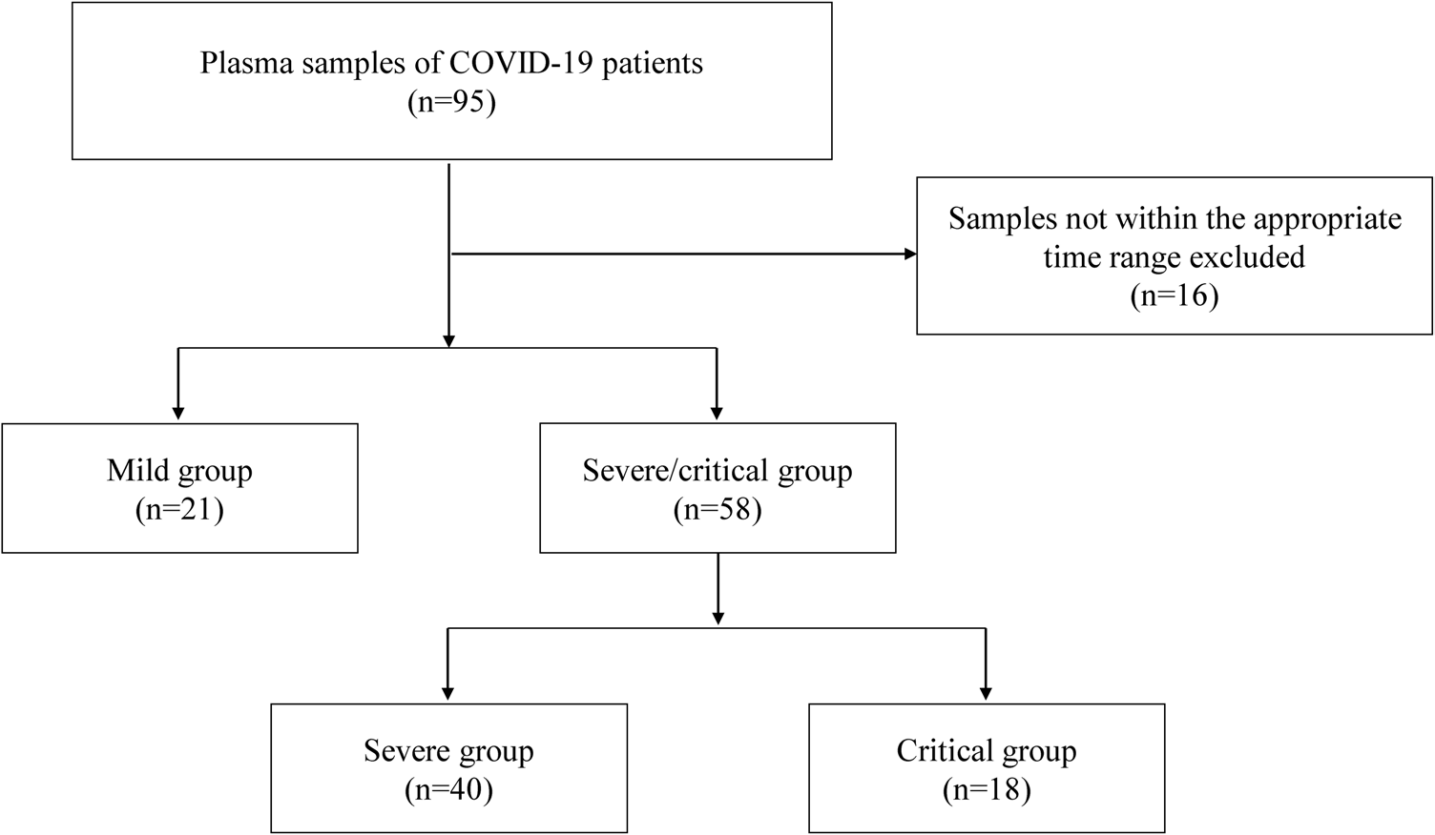
Study population flow diagram.

A total of 79 participants were included in the study. They were divided into two groups, the mild group (n = 21) and the severe/critical group (n = 58), as shown in Fig. 1. Table 1 presents the baseline characteristics and outcomes of the participants. And the original data are available in Tables S1 and S2.

**Table 1:** Baseline characters and clinical outcomes of participants.

	Mild group (n = 21)	Severe/critical group (n = 58)
Age, median (IQR), y	35 (25, 40)	82 (73, 91)
Gender		
Male, n (%)	6 (28.57)	47 (81.03)
Female, n (%)	15 (71.43)	11 (18.97)
Basic disease^a^		
Hypertension, n (%)	2 (9.52)	37 (63.79)
Cardiovascular disease, n (%)	0 (0)	24 (41.38)
Respiratory disease, n (%)	0 (0)	6 (10.34)
Diabetes, n (%)	0 (0)	12 (20.69)
Cancer, n (%)	0 (0)	6 (10.34)
Kidney disease, n (%)	0 (0)	4 (6.90)
Liver disease, n (%)	0 (0)	2 (3.45)
None, n (%)	19 (90.48)	7 (12.07)
Smoking history		
Yes, n (%)	0 (0)	9 (15.52)
No, n (%)	21 (100.00)	49 (84.48)
Allergic history		
Yes, n (%)	3 (14.29)	3 (5.17)
No, n (%)	18 (85.71)	54 (93.10)
Other^b^, n (%)	0 (0)	1 (1.73)
Previous infection history		
Yes, n (%)	0 (0)	0 (0)
No, n (%)	21 (100.00)	58 (100.00)
Highest temperature over the course of the disease		
< 37.3 °C, n (%)	2 (9.52)	1 (1.72)
37.3—38.4 °C, n (%)	5 (23.81)	12 (20.69)
38.5—40.0 °C, n (%)	10 (47.62)	44 (75.86)
> 40.0 °C, n (%)	1 (4.76)	1 (1.72)
Unknown, n (%)	3 (14.29)	0 (0)
Vaccination status		
Unvaccinated, n (%)	0 (0)	37 (63.79)
Complete partial basic vaccination, n (%)	0 (0)	11 (18.97)
Complete whole basic vaccination, n (%)	3 (14.29)	10 (17.24)
Complete booster vaccination, n (%)	18 (85.71)	0 (0)
Vaccine type		
Inactivated virus vaccine, n (%)	18 (85.71)	17 (29.31)
Recombinant protein vaccine, n (%)	3 (14.29)	0 (0)
Adenovirus vaccine, n (%)	0 (0)	1 (1.72)
Unknown, n (%)	0 (0)	3 (5.17)
Patient’s outcome		
Recovered, n (%)	21 (100.00)	44 (75.86)
Dead, n (%)	0 (0)	14 (24.14)

*IQR* interquartile range.

^a^Multiple basic diseases may exist simultaneously.

^b^Patient’s allergy history is unknown.

### RBD IgG antibody

**Figure 2:**
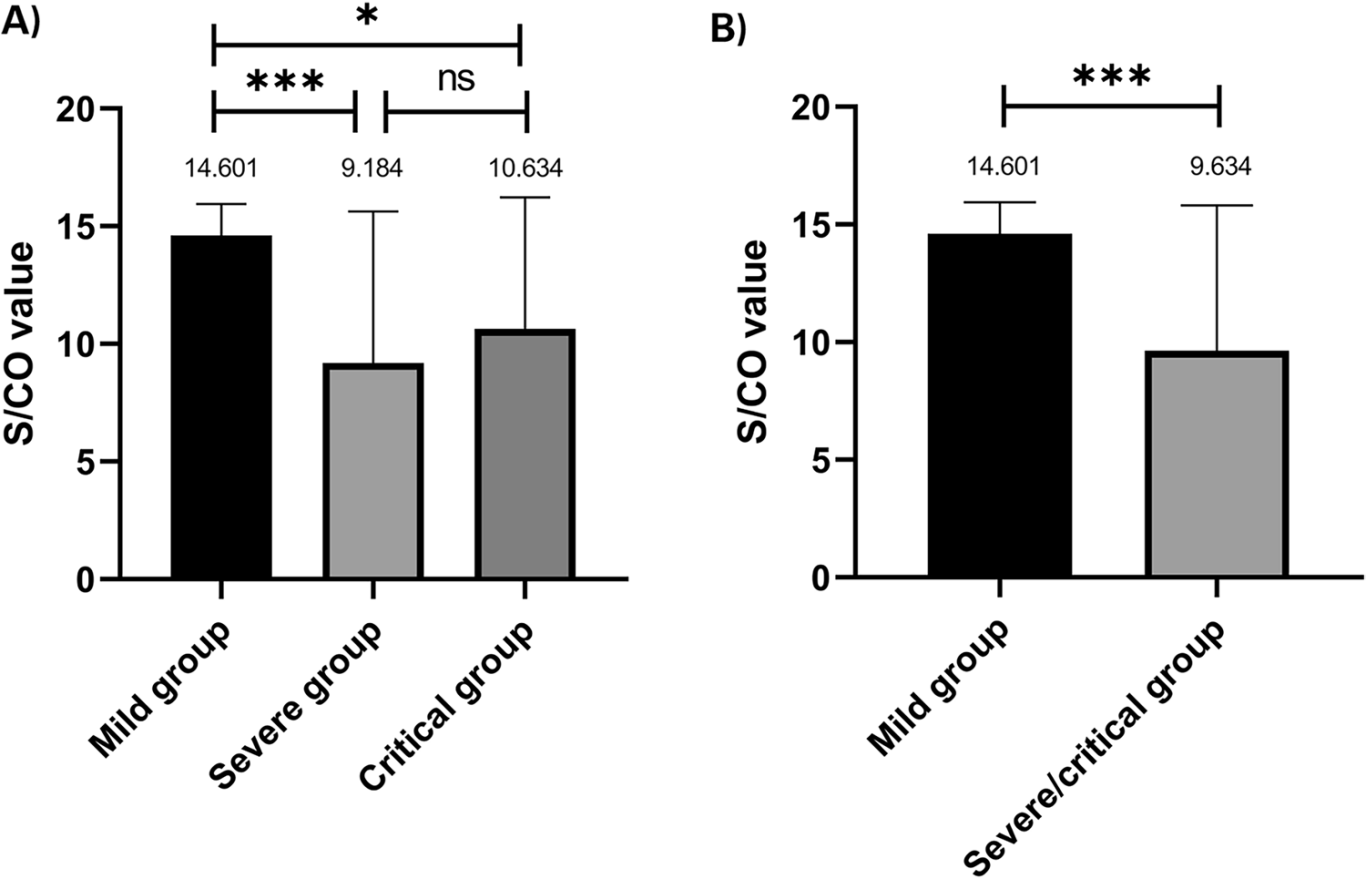
Comparison of the ELISA results. (**A**) The S/CO value of the mild group was significantly higher than that of the severe/critical group (*P* = 0.00049). (**B**) There was no difference between two subgroups (*P* > 0.05). Values above the symbols denote mean concentration. *P < 0.05, **P < 0.01, ***P < 0.001, and ns = no significance.

We compared the S/CO values of RBD (Omicron BA.4/5) IgG antibody between the two groups (Fig. 2). The difference between the two groups was found to be significant (*P* = 0.00049, Fig. 2B); the average S/CO values in the mild group were approximately 1.5-folds higher than those in the severe/critical group. Regarding the subgroups, there was no difference between severe and critical patients (*P* > 0.05, Fig. 2A).

### Correlation analysis

**Figure 3:**
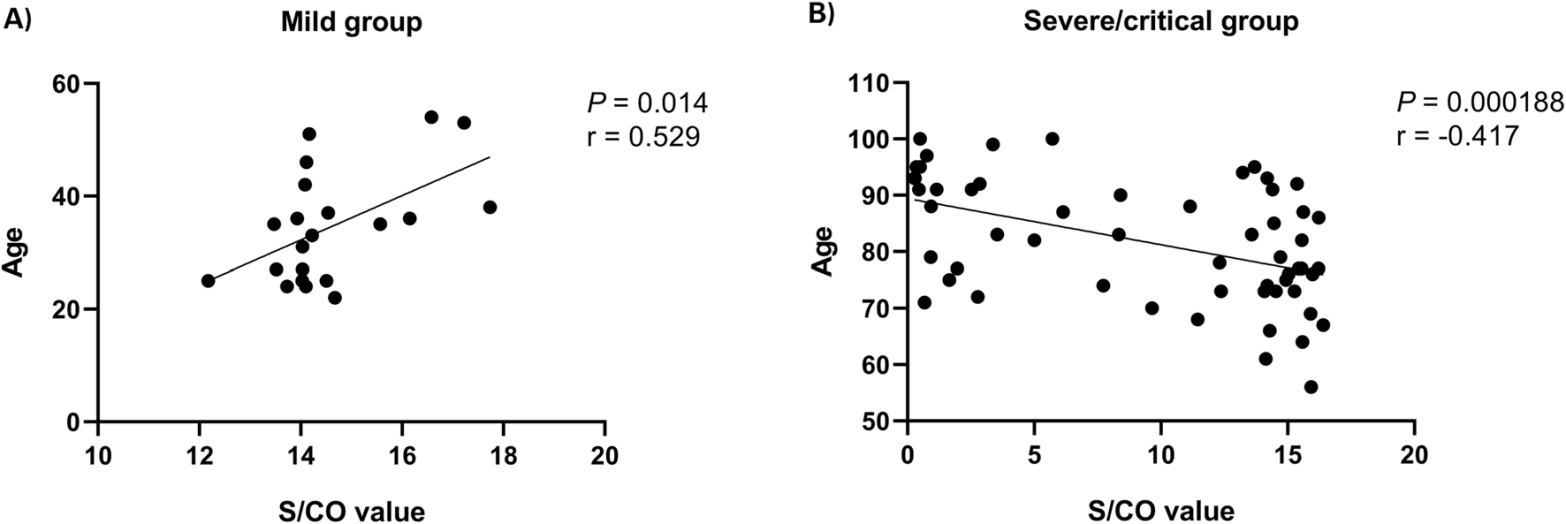
Correlation between ELISA results and ages. ELISA results were the S/CO values with 1:160 dilution. The fitted curve of S/CO values and ages in (**A**) the mild groupand (**B**) the severe/critical group.

In the two groups, we separately explored the association between plasma RBD (Omicron BA.4/5) IgG antibody levels and baseline characters or patients’ outcomes (Table 2). The results showed there existed a moderate association between plasma antibody levels and age (Fig. 3). In the mild group, plasma antibody levels were associated with basic diseases (*P* = 0.00654, eta^2^ = 0.329). And there was a correlation between plasma antibody levels and patients’ vaccination history, with higher antibody levels in vaccinated patients (*P* = 0.000286, eta^2^ = 0.211). Subsequent analysis of the patients' specific vaccination status yielded consistent results (*P* = 0.00147, eta^2^ = 0.211). No associations were found with other variables.

**Table 2:** Correlation analysis’ results of COVID-19 patients.

	Mild group (n = 21)	Severe/critical group (n = 58)
Age		
* P* value	0.014	0.000188
Correlation coefficient r^a^	0.529	− 0.471
Gender		
* P* value	0.274	0.791
Eta^2b^	–	–
Basic disease		
* P* value	0.00654	0.341
Eta^2^	0.329	–
Smoking history		
* P* value	–	0.172
Eta^2^	–	–
Allergic history		
* P* value	0.321	0.381
Eta^2^	–	–
Previous infection history		
* P* value	–	–
Eta^2^	–	–
Highest temperature over the course of the disease		
* P* value	0.520	0.086
Eta^2^	–	–
Vaccination history		
* P* value	–	0.000286
Eta^2^	–	0.211
Vaccination status		
* P* value	0.316	0.00147
Eta^2^	–	0.211
Patient’s outcome		
* P* value	–	0.701
Eta^2^	–	–

^a^In correlation analysis, the r refers to the correlation coefficient in continuous variables. R > 0, positive correlation; r < 0, negative correlation. ∣R∣ < 0.4, weak correlation; 0.4 ≤ ∣r∣ < 0.6, medium correlation; ∣r∣ ≥ 0.6, strong correlation.

^b^In correlation analysis, the eta value refers to the correlation coefficient in categorical variables. Eta^2^ < 0.06, weak correlation; 0.06 ≤ eta^2^ < 0.16, medium correlation; eta^2^ ≥ 0.16, strong correlation.

Based on the aforementioned analysis results and literature reports, we took age, gender, basic disease, and vaccination status as the independent variables, while plasma antibody level was used as the dependent variable of multivariate analysis. Multiple linear regression analysis was performed in the severe/critical group (Table 3). The results showed there was a linear relationship between age or vaccination status and plasma antibody level in severe/critical patients (*P* = 0.000807; *P* = 0.00386).

**Table 3:** Results of Multiple linear regression analysis.

Independent variable	Coefficient B	Standardized error	Standardized coefficient B	*P* value
Constant	25.443	6.055		
Age	− 0.231	0.065	− 0.402	0.000807
Gender	0.397	1.760	0.025	0.822
Basic disease	− 1.450	2.097	− 0.077	0.492
Vaccination status	2.760	0.913	0.347	0.00386

## Discussion

With the virulence of SARS-CoV-2 had gradually diminished, China declared the end of its containment measures at the end of 2022. Owing to stringent domestic controls, there had been no widespread infections nationwide in China. Following the removal of relevant containment measures, numerous individuals infected with the current circulating strain emerged. Due to its weakened virulence, the majority of infected individuals experienced mild symptoms and could recover within one to two weeks after infection, but for older infected people, they might develop severe or critical illness. In this study, we found the plasma antibody level of the severe/critical group was significantly lower than that of the mild group. We thought this difference may be caused by the age of the participants and found there was indeed an association between plasma antibody levels and age in SARS-CoV-2 infectors. Previous reports suggest that older patients exhibit a reduced humoral immune response to vaccination, lower peak antibody titers, and a more rapid decline compared to younger patients^8–11^. Clemens A. Schmitt et al.^6^ reported COVID-19 brought bigger influence in the elderly based on cellular senescence. Furthermore, Parker et al.'s investigation revealed that individuals aged 41–60 years exhibited higher plasma antibody levels than other age groups^12^. Our finding was consistent with these reported studies. It was a negative correlation between age and plasma antibody levels in our severe/critical group. That means in severe and critical patients, older age is associated with an increased risk. Besides, age was positive correlated with antibody levels in the mild group in this study. Combining the results of the two groups, there may be an age interval where the correlation between age and antibody levels changes from positive to negative as patients age.

Strong epidemiological evidence exists that sex is an important biologic variable in immunity^7^. Some data demonstrate female immune system may generate stronger antibody responses^13–16^. Whether gender differences in the humoral immune response occur in COVID-19 remains unanswered. In our study, it seemed that plasma antibody levels were not associated with gender. But it could be observed from Table 1 that the proportion of female patients was lower than that of male patients. More studies with larger sample sizes are needed to explore this association.

In addition to the factors above, we also found there existed a strong correlation between vaccination status and antibody levels in severe/critical patients. This suggests vaccination is meaningful for improving antibody levels and combating COVID-19.

There are several limitations to our study. These include the relatively small sample size, the lack of data on patients at younger ages, and the lack of clinical testing data. Besides, most patients in the mild group doesn’t have basic disease, expect two individuals, which may influence the analysis results of this part. The host immune response is complex, and factors such as vaccine type, vaccination time, sample collection time, genetic factors, therapeutic intervention, and others may affect antibody levels. Most of the participants were vaccinated with inactivated virus vaccines (see Tables 1, S1, and S2). Unfortunately, due to the difficulties in the actual information collection process, the data of vaccination type and vaccination time of all participants could not be obtained. The severe and critical patients in this study were older, the complications were common and the treatment situation was very complex and unavoidable. Thus, we regret that we were unable to deduct the impact of these factors. However, all the data were collected based on the reality. And the current study design allows for a preliminary assessment of the factors in the severe and critical COVID-19 patients. During the study period, there were no second infections among the included individuals. Further studies are needed to confirm our findings. We are also focusing on genetic factors and will conduct studies to discuss the correlation of antibody levels in patients with their immune profiles and genes.

## Conclusion

Between the mild and severe/critical patients, the level of RBD (Omicron BA.4/5) IgG antibody was significantly different. Besides, the level of plasma antibody seemed to be moderately correlated with age, suggesting that infection in the elderly should receive more attention. And plasma antibody levels were strongly associated with vaccination status in the severe/critical patients.

## Methods

### Ethics statement

The study was approved by the Ethics Committee of the Institute of Blood Transfusion, Chinese Academy of Medical Sciences & Peking Union Medical College. All participants provided written informed consent for the collection of information and for the publication of data generated by the study. All experiments were performed in accordance with relevant guidelines and regulations.

### Participants

We collected 95 plasma samples from patients infected with SARS-CoV-2 between December 2022 and March 2023. 16 samples which were not sampled within 2–5 weeks of the onset of symptoms were excluded. The included 79 samples were divided into two groups, the mild group (n = 21) and the severe/critical group (n = 58), as depicted in Fig. 1. The mild group served as the control. Patients’ disease severity ratings were judged according to clinical grading criteria, there were 40 severe patients and 18 critical patients. Severe COVID-19 was defined as respiratory distress (≥ 30 breaths/min; in resting state, oxygen saturation of 93% or less on room air; or arterial partial pressure of oxygen (PaO_2_)/fraction of inspired oxygen (FIO_2_) of 300 or less. Critical COVID-19 was defined as respiratory failure requiring mechanical ventilation; shock; or other organ failure (apart from lung) requiring intensive care unit (ICU) monitoring. The baseline characters and patients’ outcomes of the participants is shown in Table 1.

### Enzyme-linked immunosorbent assay (ELISA)

Antibodies were detected using the Vazyme SARS-CoV-2 RBD (Omicron BA.4/5) IgG Antibody Detection Kit (Nanjing Vazyme Biotechnology Co., Cat.: DD3142-01) according to the manufacturer instructions. Microporous plates were precoated with SARS-CoV-2 RBD protein. The samples were diluted at 1: 160, and added to microporous plates and incubated at 37 °C for 1 h. The microporous plates were washed five times. Horseradish peroxidase labeled mouse-anti-human IgG monoclonal antibody was added and incubated at 37 °C for 30 min. After incubation, the plate was washed five times, substrate buffer containing hydrogen peroxide and tetramethylbenzidine (TMB) was added to the wells and incubated at room temperature for 15 min. Then, the termination solution was added, and a spectrophotometer was used to detect the optical density (OD) value of the wells under a dual-wavelength excitation light of 450 and 630 nm. Positive and negative results were determined by sample/cut off (S/CO) values which were calculated according to the manufacturer’s protocol.

### Statistical analysis

Statistical analysis was conducted using GraphPad Prism 8 and SPSS 25. All data in the figures is presented as mean ± SD. The results were evaluated using the Kolmogorov–Smirnov test, the independent-sample t test and ANOVA test. In the correlation analysis, the F test in ANOVA was used for categorical variables. And in the quantitative variables, Pearson correlation analysis was used for normally distributed continuous variables, while Spearman correlation analysis was used for non-normally distributed continuous variables. The multivariate analysis was used to determine the influence of different factors on each other in the correlation analysis. *P* < 0.05 was considered statistically significant (*P < 0.05, **P < 0.01, and ***P < 0.001).

### Ethical approval

The study was approved by the Ethics Committee of the Institute of Blood Transfusion, Chinese Academy of Medical Sciences & Peking Union Medical College. Written informed consent was obtained from each study participant.

### Supplementary Information

Supplementary Information 1.

Supplementary Information 2.

Supplementary Information 3.

Supplementary Information 4.

## Data Availability

All data generated or analyzed during this study are included in this article/Supplementary material.
